# The elicitation of a systemic resistance by *Pseudomonas putida *BTP1 in tomato involves the stimulation of two lipoxygenase isoforms

**DOI:** 10.1186/1471-2229-11-29

**Published:** 2011-02-04

**Authors:** Martin Mariutto, Francéline Duby, Akram Adam, Charlotte Bureau, Marie-Laure Fauconnier, Marc Ongena, Philippe Thonart, Jacques Dommes

**Affiliations:** 1 Laboratory of Plant Molecular Biology and Biotechnology, Faculty of Sciences, Department of Life Sciences, University of Liège, Boulevard du Rectorat, 27, Liège, Belgium; 2 Walloon Centre of Industrial Biology, University of Liège, Boulevard du Rectorat, 29, Liège, Belgium; 3 Walloon Centre of Industrial Biology, Gembloux Agro-Bio Tech, University of Liège, Passage des Déportés, 2, Gembloux, Belgium; 4 Plant Biology Unit, Gembloux Agro-Bio Tech, University of Liège, Avenue de la Faculté, 2A, Gembloux, Belgium

## Abstract

**Background:**

Some non-pathogenic rhizobacteria called Plant Growth Promoting Rhizobacteria (PGPR) possess the capacity to induce in plant defense mechanisms effective against pathogens. Precedent studies showed the ability of *Pseudomonas putida *BTP1 to induce PGPR-mediated resistance, termed ISR (Induced Systemic Resistance), in different plant species. Despite extensive works, molecular defense mechanisms involved in ISR are less well understood that in the case of pathogen induced systemic acquired resistance.

**Results:**

We analyzed the activities of phenylalanine ammonia-lyase (PAL) and lipoxygenase (LOX), key enzymes of the phenylpropanoid and oxylipin pathways respectively, in tomato treated or not with *P*. *putida *BTP1. The bacterial treatment did not stimulate PAL activity and linoleate-consuming LOX activities. Linolenate-consuming LOX activity, on the contrary, was significantly stimulated in *P. putida *BTP1-inoculated plants before and two days after infection by *B. cinerea*. This stimulation is due to the increase of transcription level of two isoforms of LOX: *TomLoxD *and *TomLoxF*, a newly identified LOX gene. We showed that recombinant TomLOXF preferentially consumes linolenic acid and produces 13-derivative of fatty acids. After challenging with *B. cinerea*, the increase of transcription of these two LOX genes and higher linolenic acid-consuming LOX activity were associated with a more rapid accumulation of free 13-hydroperoxy-octadecatrienoic and 13-hydroxy-octadecatrienoic acids, two antifungal oxylipins, in bacterized plants.

**Conclusion:**

In addition to the discovery of a new LOX gene in tomato, this work is the first to show differential induction of LOX isozymes and a more rapid accumulation of 13-hydroperoxy-octadecatrienoic and 13-hydroxy-octadecatrienoic acids in rhizobacteria mediated-induced systemic resistance.

## Background

Plants possess a large variety of defense mechanisms to prevent and fight pathogen attacks: their structural and chemical, preformed and inducible defense mechanisms limit the infection. When an avirulent pathogen meets a resistant plant, cells located around the infection site die within a few hours of contact. This phenomenon, called hypersensitive response, may cause damages to the pathogen and also leads to a mobile signal that will induce defense mechanisms in uninfected parts of the plant [1]. In a zone of some millimeters around the hypersensitive response site, cells develop the local acquired resistance [2], characterized by the reinforcement of the cell wall, synthesis of antimicrobial phytoalexins, and expression of pathogenesis-related (*Pr*) genes [3]. At distant sites in the plant, systemic acquired resistance (SAR) is induced [4]. This resistance is associated with an accumulation of salicylic acid, *Pr*genes expression and stimulation of many defense pathways [5].

Other kinds of micro-organisms can induce a resistance in plants against diseases: the non-pathogenic rhizobacteria, referred to as plant growth promoting rhizobacteria (PGPR), can protect plants against pathogens. PGPR can affect pest population by antibiosis, nutrient competition or niche exclusion [6]. In addition to these direct antagonisms, rhizobacteria can induce a systemic resistance that makes the plant more resistant to a future pathogen attack. This long lasting, broad spectrum resistance, called induced systemic resistance (ISR) [7], is phenotypically similar to SAR, but molecular events leading to its induction are different. ISR is not associated with an increase of salicylic acid [8] neither other hormones but needs the perception to jasmonate and ethylene [9]. Transduction pathway of ISR and SAR are different but both need the regulatory protein NPR1 [10]. Downstream, the two pathways differ again because *Pr *genes are not expressed in ISR [9].

Despite extensive work, the protective mechanisms involved in ISR are less well understood than those involved in SAR. In many pathosystems, two defense pathways are generally associated with the enhanced protection level conferred by ISR: the phenylpropanoid pathway and the oxylipin pathway. *Bacillus cereus *B101R and *B. subtilis *AF 1 induce lipoxygenase (LOX) activity in tomato [11] and groundnut [12] respectively. This enzyme is a dioxygenase that transforms poly-unsaturated fatty acids into hydroperoxides. It catalyses the first step of the oxylipin pathway. In tomato as in other angiosperms, lipoxygenase is encoded by a multigene family. *TomLoxA*, *TomLoxB*, and *TomLoxE *are expressed principally in fruits during ripening [13,14]. *TomLoxC *is expressed in fruits and leaves, and its products are converted into volatile aldehydes and alcohols [14] responsible for the characteristic aroma of tomato plants [15]. *TomLoxD *expression is stimulated by wounding, jasmonate, and systemin. This enzyme leads to the synthesis of defense compounds called octadecanoids [16]. Hydroperoxides are consumed by different enzymes to generate oxylipins, among which one finds signal molecules such as jasmonic acid, aldehydes, and defense metabolites such as hexenal (a volatile), colneleic acid, and colnelenic acid [17].

In *Pseudomonas fluorescens *WCS417r-inoculated carnation, phytoalexin synthesis is stimulated [7]. In pea treated with *Bacillus pumilus *SE34 [18], macroscopic protection has been linked to reinforcement of the cell wall by deposition of callose, pectin, and other phenolic compounds. *Trichoderma asperellum *T-203 protects cucumber against *Pseudomonas syringae *pv. *lachrymans *by inducing phytoalexin synthesis through stimulation of the expression of genes coding for phenylalanine ammonia-lyase (PAL) and hydroperoxide lyase, an enzyme of the oxylipin pathway [19]. Lignin and certain phytoalexins are produced via the phenylpropanoid pathway. The first step of this pathway is catalyzed by PAL, which converts phenylalanine to cinnamic acid, the precursor of lignin, salicylic acid, some pigments such as anthocyanidins, condensed tannins, and phytoalexin phenylpropanoids [20]. Enzyme stimulation as part of ISR is generally effective after pathogen infection [9].

Previous studies have shown that *P. putida *BTP1, a PGPR strain isolated from a barley field [21], can induce ISR against *Pythium aphanidermatum *[22] in cucumber and against *Botrytis cinerea *in bean [23] and tomato [24]. In cucumber, the protection conferred by *P. putida *BTP1 is associated with the accumulation, after pathogen inoculation, of fungitoxic phenolics that can be viewed as phytoalexins. ISR in bean is characterized by enhanced levels of LOX activity and poly-unsaturated fatty acids before pathogen challenge, and by stimulation of hydroperoxide lyase activity and volatile oxylipins production after pathogen challenge. PAL activity, however, is not stimulated. LOX activity has been shown to be stimulated in *P. putida *BTP1-treated tomato plants after infection, but as these experiments were done on detached leaves and as LOX activity is stimulated by wounding [16], the results might be different for whole plants.

In this work, ISR induced by *P. putida *BTP1 was studied in whole tomato plants. We showed that the PAL activity is not induced by the ISR, contrary to the LOX activity. As LOX is encoded by a multigene family, we measured the expression levels of five genes to identify which isoforms might contribute to this increase. We showed that only two genes participated to this stimulation: *TomLoxD *and a newly identified gene, *TomLoxF*. We cloned and expressed *TomLoxF *in bacteria to characterize its protein, and showed that the recombinant TomLOXF preferentially consumed linolenic acid and introduced oxygen onto the 13^th ^carbon of the fatty acid. Finally, we confirmed our results by analyzing the accumulation of the products of TomLOXF in the plant, and showed that the 13-hydroperoxy-octadecatrienoic acid and its reduced form were more abundant in bacterized plants.

## Results

### Phenylalanine ammonia-lyase is not stimulated in *P. putida *BTP1-mediated ISR

**Figure 1:**
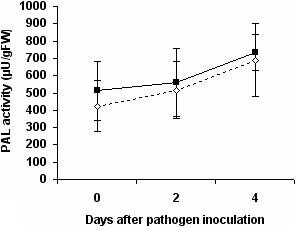
**Time course of phenylalanine ammonia-lyase (PAL) activity**. The activity was measured in the leaves of control (dotted line) and *P. putida *BTP1- treated (continuous line) tomato plants before (0), two days (+2), and four days (+4) after challenge with *B. cinerea*. Statistical analysis (Student's T test, α = 0.05) revealed that differences between control and bacterized plants, at the same infection time, were not significant. Data are means and standard deviations calculated from three measurements on two enzyme extracts.

PAL activity was quantified before and after pathogen inoculation in control and bacterized plants, in order to assess whether this pathway contributes to the enhanced protection level associated with ISR. No significant difference was detected between control and *P. putida *BTP1-treated plants, either before pathogen challenge or two or four days after infection (by *B. cinerea*) (Figure 1).

### *P. putida *BTP1 induces lipoxygenase activity

**Figure 2:**
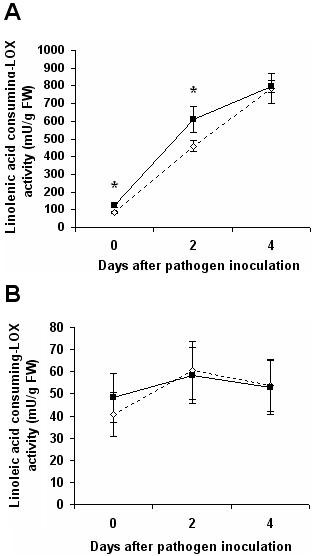
**Time course of consumption of linolenic (A) and linoleic (B) acids by LOX**. The activities were monitored in control (dotted line) and *P. putida *BTP1-treated (continuous line) tomato leaves. Samples were collected before (0), two days (+2), and four days (+4) after inoculation of *B. cinerea*. Stars (*) indicate statistically significant differences between control and treated plants (Student's t test, α = 0,01). Data are means and standard deviations calculated from three measurements on two enzyme extracts.

The linolenic-acid- and linoleic-acid-consuming LOX activities of treated and untreated plants were monitored to determine if they are stimulated by *P. putida *BTP1. Before infection, as shown in Figure 2, consumption of linolenic acid by LOX was higher in bacterized plants than in control plants. The activity increased in response to infection and two days after pathogen inoculation, it remained higher in the treated plants. After four days of infection, the activity difference was no longer significant (Student's T test, α = 0,01) (Figure 2a). In contrast, *P. putida BTP1*-treated and control plants showed no significant difference in linoleic acid consumption either before or after inoculation of *B. cinerea *(Figure 2b). The linoleic-acid-consuming LOX activity thus did not seem to be influenced by infection or by ISR.

### Expression of *TomLox *genes in response to *P. putida *BTP1 treatment

**Figure 3:**
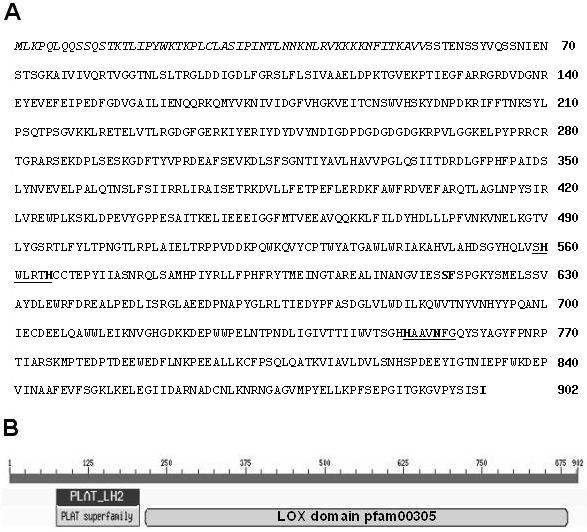
**Analysis of the amino acid sequence of the tomato lipoxygenase F**. A: Deduced amino acid sequence of TomLOXF. The conserved motives are underlined, and the conserved amino acid residues involved in LOX iron binding, enzymatic activity, and regio-specificity are in bold. The characters in italics indicate the putative chloroplastic transit peptide identified with ChloroP1.1. Numbers on the righ indicate the position occupied in the protein sequence by the last amino acid of the line. B: Schematic representation of the PLAT_LH2 domain and of the LOX domain pfam00305 identified in TomLOXF by the NCBI Conserved Domain Search program.

**Figure 4:**
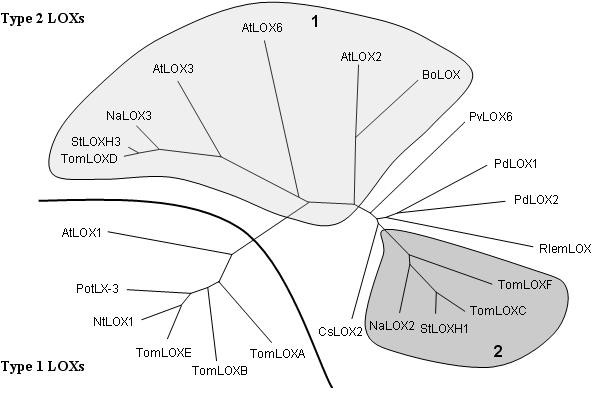
**Phylogenetic tree of various lipoxygenases from plants**. Sequence relatedness between the deduced amino acid sequence of TomLOXF and sequences of LOX proteins of diverse plants was analyzed with ClustaW2 by the neighbor-joining method, and visualized with the TreeDyn program. Accession numbers for the LOX amino acid sequences used to construct the tree are: *Lycopersicon esculentum *TomLOXA [GenBank: AAA53184], TomLOXB [GenBank: AAA53183], TomLOXC [GenBank: AAB65766], TomLOXD [GenBank: AAB65767], TomLOXE [GenBank: AAG21691]; *Solanum tuberosum *StLOXH1 [GenBank: CAA65268], StLOXH3 [GenBank: CAA65269], PotLX-3 [GenBank: AAB67865]; *Nicotiana attenuata *NaLOX2 [GenBank: AAP83137], NaLOX3 [GenBank: AAP83138]; *Nicotiana tabacum *NtLOX1 [GenBank: CAA58859]; Camellia sinensis CsLOX2 [GenBank: ACJ54281]; *Populus deltoids *PdLOX1 [GenBank: AAZ57444], PdLOX2 [GenBank: AAZ57445]; *Phaseolus vulgaris *PvLOX6 [GenBank: ABM88259]; *Citrus jambhiri *RlemLOX [GenBank: BAB84352]; *Arabidopsis thaliana *AtLOX1 [GenBank: NP_175900], AtLOX2 [GenBank: AAL32689], AtLOX3 [GenBank: CAB56692], AtLOX6 [GenBank: CAG38328]; Brassica oleracea BoLOX [GenBank: ABO32545]. Type 1 and Type 2 respectively indicate LOX proteins involved in jasmonic acid or C6 volatile production.

To determine which LOX isozyme(s) might be involved in the LOX activity increase during ISR, we analyzed *TomLox *gene expression. During our attempts to clone a *TomLoxC *cDNA probe, a 500-bp RT-PCR product was amplified from total RNA extracted from methyljasmonate-treated tomato leaves. Sequencing of this product revealed that the corresponding cDNA shares 82% similarity with *TomLoxC*. Gene-specific primers for 3' and 5' rapid amplification of cDNA ends (RACE) were synthesized on the basis of this sequence. The 5'RACE and 3'RACE products were amplified from the RNA of tomato leaves treated with methyljasmonate and from the RNA of tomato leaves treated with *P. putida *BTP1. The sequences of all the overlapping RACE products were strictly identical (apart from a slight variability observed at the level of the 5'- and 3'-UTRs), and a full-length 2837-bp cDNA, called *TomLoxF*, was identified (GenBank: FJ617476) (Figure 3). This cDNA sequence was found to harbor a complete 2,709-bp open reading frame flanked by a 40-bp 5'-UTR and a 88-bp 3'-UTR. The first ATG encountered from the 5'-end of the cDNA was considered to be the start codon of the open reading frame (a TAA stop codon is located in frame, 9 bp upstream from this ATG). The deduced protein sequence consists of 903 amino acid residues with a calculated molecular mass of 102.5 kDa. Blastp analysis of the deduced TomLOXF amino acid sequence showed that this protein shares 40 to 79% identity with other known plant LOX proteins and that it possesses the two domains that are typically conserved in plant lipoxygenase proteins, the PLAT_LH2 domain (positions 72-206) and the LOX domain pfam00305 (positions 215-886) (Figure 3). The plant lipoxygenases most closely related to TomLOXF are *Solanum tuberosum *StLOXH1 [25], *Nicotiana attenuata *NaLOX2 [26], and *Lycopersicon esculentum *TomLOXC [16], sharing respectively 79%, 78%, and 76% identity with TomLOXF at the amino acid level. In contrast, the predicted amino acid sequence of TomLOXF displays only 40-49% identity to other identified tomato lipoxygenases. TomLOXF contains known LOX motifs harboring all the amino acid residues conserved among plant LOX proteins (His560, His565, His752, Asn756 and Ile902, Figure 3) and involved in iron binding and enzyme catalytic activity [27]. Furthermore, TomLOXF possesses the conserved Ser/Phe motif (S617 and F618) occurring at the bottom of the substrate-binding pocket of nearly all plant LOX enzymes that introduce dioxygen onto the 13^th ^carbon of the fatty acid (13-LOX) and determining their regio-specificity [28,29]. A phylogenetic analysis was performed in order to determine the proximity of TomLOXF to other plant LOX proteins. Multiple sequence alignments were done and an unrooted phylogenetic tree was constructed (Figure 4). According to the classification of Feussner and Wasternack [28], the tree could be divided into two major groups. The first group includes the type 1 lipoxygenases, which are enzymes harboring no transit peptide and sharing high within-group sequence similarity. The second group includes the type 2 lipoxygenases, which carry a putative chloroplast transit peptide sequence and share only moderate overall within-group sequence similarity. To date, the type-2 LOX proteins all belong to the 13-LOX subfamily [28,29]. This group can be further divided into two subgroups. The first includes, among others, AtLOX2 [30], BoLOX [31], NaLOX3 [26], TomLOXD [16], and StLOXH3 [25], enzymes shown to be involved in the wound-induced biosynthesis of jasmonic acid. The second group includes StLOXH1 [25] and TomLOXC [16], two LOX isoforms playing a key role in the generation of fatty-acid-derived short-chain volatiles [14,32]. The topology of the phylogenetic tree clearly shows that TomLOXF belongs to the type-2 LOX group and that it is closely related to enzymes producing hydroperoxides consumed preferentially by hydroperoxide lyase, a C6-volatile-producing enzyme. Prediction of the subcellular localization of the TomLOXF protein was done by means of four different programs. The iPSORT program predicted a mitochondrial localization, whereas the presence of a transit peptide for chloroplast targeting was predicted by the TargetP1.1, WoLFPSORT, and ChloroP1.1 programs. ChloroP1.1 identified a 54-residue chloroplast transit peptide at the N- terminus of the TomLOXF protein. This N-extension of the sequence shows some features typical of a chloroplast sorting signal [33], including a high content in hydrophilic amino acid residues (18.5% Lys, 16.7% Ser and Thr) and a very low content in acidic residues (no Asp or Glu). In this group, AtLOX2, TomLOXC, TomLOXD, StLOXH1, StLOXH3, and PvLOX6 have been demonstrated to be actively imported into or localized within the chloroplast [14,16,32,34-36]. On the basis of these observations, it is likely that TomLOXF also encodes a chloroplast-targeted LOX.

**Figure 5:**
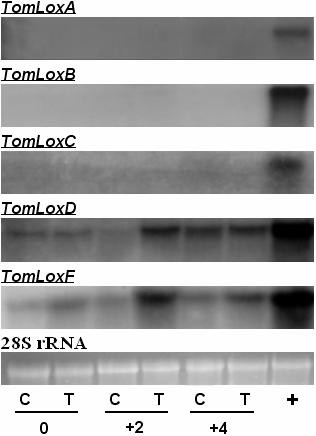
**Comparison of expression levels of five *TomLox *genes (A, B, C, D, and F)**. The expression levels were compared between control (C) and *P. putida *BTP1-treated (T) plants. Samples were collected before (0), two days (+2), and four days (+4) after pathogen inoculation. + represents transcripts of the positive control: for *TomLoxA*, *TomLoxB*, and *TomLoxC*, the positive control was RNA extracted from breaker-stage fruit, for *TomLoxD *and *TomLoxF*, the positive control was RNA extracted from methyljasmonate-treated plants. Total RNA was extracted from leaves and 20-μg samples were subjected to RNA-blot analysis, except for the positive controls for *TomLoxA*, *TomLoxB*, and *TomLoxC*, for which 2 μg was loaded. Transcripts were hybridized with denatured cDNA-specific probes. Quantification of loading of each sample RNA was done by measuring the U.V. fluorescence of ethidium-bromide-stained 28 S rRNA. Loading was found to vary by 20% at most between samples.

As LOX activity was induced by the bacterial treatment, the expression of each gene in response to *P*. *putida *BTP1 treatment was analyzed at transcript level in order to determine the relative contribution of the various isoforms to activity increase. Before infection, *TomLoxA*, *TomLoxB*, and *TomLoxC *transcripts were barely detected in leaves of control and treated tomato plants (Figure 5). These genes were found not to be upregulated upon pathogen attack and the transcript level was similar for control and treated plants. The *TomLoxD *and *TomLoxF *genes displayed a different expression profile: basal-level expression before infection but clearly increased expression upon pathogen challenge, the increase being more pronounced in plants bacterized beforehand with *P. putida *BTP1 than in control plants. This differential stimulation of the transcription level in control and treated plants was transient in the case of *TomLoxD*, since similar amounts of transcripts were found to have accumulated in leaves from both kinds of plants 96 hours after infection by *B. cinerea*. Stimulation of the *TomLoxF *gene in bacterized plants appeared more consistent, since the transcript level remained slightly higher than in control leaves four days post infection.

### TOMLOXF uses linolenic acid as substrate and exhibits 13-LOX activity

**Figure 6:**
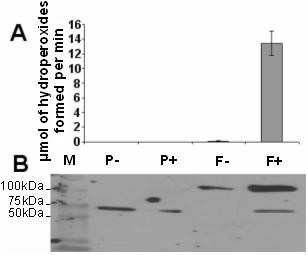
**Evaluation of recombinant TomLOXF produced in *E. coli***. We verified the production and activity of the recombinant TomLOXF through LOX activity assay (A) and SDS-PAGE and Western blotting with an anti-His-tag antibody (B). Four clones were tested: two clones containing pET28-a plasmid without the *TomLoxF *insert (P) and two clones containing pET28-a with the *TomLoxF *insert (F), induced (+) or not (-) by IPTG. M: Page Ruler Plus Prestained Protein Ladder (Fermentas).

**Figure 7:**
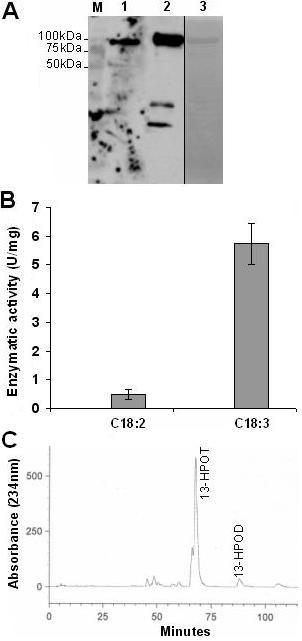
**Characterisation of TomLOXF**. **A**. The detection of his-tagged proteins was realized on Western blot with an anti-His-tag antibody (1 and 2) and we evaluated the purity of the protein through SDS-PAGE and Coomassie blue staining (3). 1: Page Ruler Plus Prestained Protein Ladder (Fermentas), 1: total proteins extracted from TomLOXF-expressing *E. coli*, 2 and 3: partially-purified protein extracted from TomLOXF-expressing clone by nickel affinity chromatography.**B**. LOX activity was evaluated on partially-purified recombinant TomLOXF with linoleic (C18:2) and linolenic (C18:3) acids as substrate. Reaction was performed at pH 6.0, room temperature. **C**. Linolenic and linoleic acids were both incubated with extracts of *E. coli *expressing TomLOXF in oxygenated buffer. Produced hydroperoxides were separated by HPLC, and the profile of compounds absorbing at 234 nm was compared with the profile of pure 13-HPOT, 13-HPOD, 9-HPOT and 9-HPOD.

To check whether increased *TomLoxF *transcription could be partly responsible for increased linolenic acid-consuming activity, we cloned and expressed the *TomLoxF *cDNA in *E. coli*, without its choroplastic peptide signal, but with a poly-His tag. Total proteins were extracted by sonication and analyzed by SDS-PAGE and Western blotting with an anti-His-tag antibody (Figure 6b). This showed the presence of a ± 100 kDa protein in extracts from clones containing the *TomLoxF*. LOX activity was assayed in the total protein extracts using linolenic acid as substrate. We only detected LOX activity in the extract from the clone containing the *TomLoxF *sequence and induced by IPTG (Figure 6a). Recombinant TOMLOXF was purified by affinity chromatography and detected through SDS-PAGE and Western blotting with an anti-His-tag antibody (Figure 7a). The purity of the purified protein was checked by SDS-PAGE and Coomassie blue staining. It showed the presence of some contaminating proteins. The activity of the semi-purified TOMLOXF was evaluated using either linoleic acid or linolenic acid as substrate. Partially-purified TOMLOXF showed a higher activity on linolenic acid than on linoleic acid (activities of 5,74 U/mg of total protein and 0,48 U/mg respectively) (Figure 7b).

Depending on their regiospecificity, LOX enzymes can introduce the oxygen at the 9^th ^or 13^th ^position of linoleic and linolenic acids. To determine the regiospecificity of partially purified TOMLOXF, we first monitored its pH activity profile using linolenic acid as substrate (data not shown) to optimize the pH of the reaction buffer. Partially purified TOMLOXF had an activity optimum of pH 6.0. TOMLOXF was then incubated at this pH with its two substrates, in combination or separatly, and the reaction products were analyzed by HPLC. In all cases only 13-derivatives (13-HPOT and 13-HPOD) of fatty acids were detected suggesting that TomLOXF is a 13-LOX (Figure 7c).

On the basis of these similarities, it was hypothesized that TomLOXD is a linolenate-consuming lipoxygenase [16]. To confirm this activity, we also cloned and expressed the *TomLoxD *cDNA to obtain recombinant His-tagged TomLOXD protein. As the chloroplastic signal peptide can provoke some problems during the production in bacteria, we determined it with the "ChloroP" bioinformatics program and it was not included in the sequence cloned in pET-28a plasmid. Unfortunately, no enzymatic activity was detected whatever the position of the His's-tag (amino terminal or carboxy terminal). We hypothesized that the chloroplastic signal peptide determined by the program was maybe too long: it indeed contained a part (5 amino acids) of a beta barrel probably involved in substrate binding. We aligned the sequences of TomLOXF and TomLOXD and determined manually the signal peptide of TomLOXD. We cloned once again the cDNA without the sequence of the signal and expressed it in *E*. *coli *BL21. We also cloned the full cDNA including the signal peptide. After induction of expression, we were not able to detect any LOX activity for any of the constructs. We also tried to express the different constructs in other strains of *E. coli*: *E. coli *C43DE3 (usually used for the production of toxic proteins), in *E. coli *HMS174DE3, and in *E. coli *KRX (autoinduction by rhamnose), but no result was obtained (data not showed).

### Treatment with *P. putida *BTP1 induces a more rapid accumulation of oxylipins

**Figure 8:**
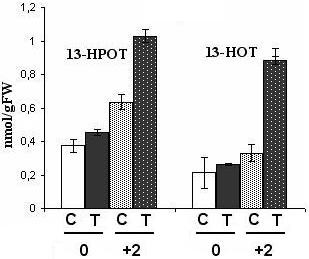
**Time course of accumulation of the free 13-HPOT and its reduced derivative, the 13-HOT**. The concentration of these two compounds was measured in control plants before infection (C0), and two days after pathogen inoculation (C2) and in *P. putida *BTP1-treated plants before infection (T0) and two days after pathogen inoculation (T2) in two independent experiments. The analysis of variance (ANOVA 1, α = 0.05) revealed that differences between control and bacterized tomatoes are significative for the 13-HPOT before *B. cinerea *inoculation, and for the 13-HOT and 13-HPOT after infection. Means and standard deviations were calculated from one measurement on two different extractions of each sample.

We also analyzed the level of two free oxylipins: the 13-hydropreoxyoctadecatrienoic acid (13-HPOT), which is produced by LOX from linolenic acid, and its reduced derivative, the 13-hydroxyoctadecatrienoic acid (13-HOT). We quantified these molecules before and two days after pathogen inoculation, where transcriptional and enzymatic differences were shown to be maximal between control and bacterized plants. As expected, we observed differences between control and treated plants: before infection, the level of free 13-HOT was only slightly higher in *P. putida *BTP1-treated plants than in control plants, but two days after pathogen inoculation it was about two fold higher in treated-plants than in control plants (Figure 8). 13-HPOT also seemed more abundant in bacterized plants after pathogen challenge.

## Discussion

Results from this study show that pre-inoculation of the rhizobacterium *P. putida *BTP1 on tomato roots protects the host plant against gray mold caused by the fungal pathogen *B. cinerea *on leaves. Previous studies carried out in cucumber [22], in bean [23] and in tomato [24] showed that *P. putida *BTP1 is not able to induce SAR. Moreover, the bacteria do not migrate to the leaves, demonstrating that the reduction of the symptoms is not caused by a direct antagonism between *P. putida *BTP1 and *B. cinerea *[24]. These observations suggest that disease reduction by *P. putida *BTP1 in indeed a case of ISR.

This resistance is not associated with stimulation of phenylalanine ammonia-lyase. Involvement of this enzyme in ISR can vary according to the plant species and pre-inoculated rhizobacterial strain. As in the case of *P. putida *BTP1, the resistance induced by *B. cereus *B101R in tomato is not characterized by stimulation of PAL [11], but in *Pseudomonas fluorescens *Pf1-treated tomatoes, resistance is associated with enhanced PAL activity after inoculation of the pathogen *P. aphanidermatum *[37]. *T. asperellum *T-203 protects cucumber against *P. syringae *pv. *lachrymans *by inducing phytoalexin synthesis through stimulation of phenylalanine ammonia-lyase expression [19]. In bean, *P. putida *BTP1 does not induce PAL activity [23], but in cucumber, the resistance conferred by this rhizobacterium is associated with the accumulation of fungitoxic phenolics that can be viewed as phytoalexins and that may be produced via the phenylpropanoid pathway [22].

Unlike PAL activity, LOX activity consuming linolenic acid is stimulated by treatment with *P. putida *BTP1. Before infection, LOX activity is higher in treated plants than in controls. It increases in response to pathogen attack, remaining higher in treated plants two days after pathogen inoculation.

We have further investigated which LOX isoforms are involved in this stimulation, and we have identified and characterized a full-length cDNA encoding a new LOX from tomato leaves: TomLOXF is the sixth lipoxygenase isoform identified in tomato. Bioinformatic analysis shows that the product of this gene belongs to the type 2 subset of LOX proteins. All the LOX proteins identified to date in this group are 13-LOX proteins, and a chloroplastic localization has been demonstrated for AtLOX2 from *Arabidopsis thaliana*, TomLOXC and TomLOXD from tomato, StLOXH1 and StLOXH3 from potato, and PvLOX6 from bean [14,16,32,34-36]. This class of LOX is known to be involved in biotic and abiotic stresses. As initial reaction, 13-LOX catalyses the insertion of molecular oxygen at position 13 of linoleic or linolenic acid.

The fatty acid hydroperoxides produced might thus be converted to jasmonic acid via the octadecanoid pathway or be metabolized via the lipoxygenase pathway by a variety of enzymes, including hydroperoxide lyase, allene oxide synthase, divinyl ether synthase, to form diverse plant-associated oxylipins such as volatile aldehydes, alcohols, divinyl ethers,... [28,38,39]. Although different branches of the pathway utilize hydroperoxides as a common substrate, recent studies tend to demonstrate that specific substrates for the enzymes of these branches are supplied by distinct LOX isoforms [40].

In *Arabidopsis thaliana*, AtLOX2 has been directly linked to the biosynthesis of jasmonic acid [34]. Similarly, silencing of *NaLox3 *in *Nicotiana attenuata *specifically suppresses JA accumulation upon injury, but does not affect the production of leaf volatiles [26]. In potato, antisense inhibition of *StLoxH3 *expression has no effect on the release of volatiles [32] or on wound-induced JA accumulation, but it drastically reduces the post injury accumulation of protease inhibitors, thereby enhancing the susceptibility of the plants to insect attack [41]. The product of *TomLoxD *is expressed mainly in response to wounding or methyl jasmonate treatment. It may also play a role as a component of the octadecanoid defense-signaling pathway, leading to the production of jasmonic acid [16], but not to the generation of volatiles [14].

On the other hand, specific depletion of *TomLoxC *in tomato has no effect on jasmonic acid biosynthesis, whereas it results in a marked reduction in the production of fatty-acid-derived C6 short-chain aldehydes and alcohols [14]. It thus seems that the prime role of TomLOXC and StLOXH1 is to supply hydroperoxide lyase with substrates for the production of C6 volatiles, but not to supply hydroperoxides to the octadecanoid pathway.

As mentioned by Feussner and Wasternack [28], phylogenetic tree analysis of the LOX multigene family might be helpful in predicting at least some biochemical features and may provide suggestions regarding physiological functions. From this analysis, it clearly appears that TomLOXD, AtLOX3, NALOX3, and StLOXH3, all similarly involved in JA biosynthesis, are closely related. On the basis of these similarities, it was suggested that TomLOXD possesses a linolenate-consuming lipoxygenase activity [16]. However this was never definitely proved. So we tried to produce recombinant His-tagged TomLOXD in *E. coli*. Unfortunately, despite numerous attempts, we were not able to produce an active TomLOXD protein to confirm this hypothesis. TomLOXD may be an inactive protein in plant tissues, but this hypothesis could be excluded as a protein close to TomLOXD (stLOX3, 84% of identity with TomLOXD) showed activity [25]. The *E. coli *expression system used here is probably not appropriate for the expression of TomLOXD.

TomLOXC and StLOXH1, key lipoxygenases specifically involved in the generation of volatiles, are grouped on another branch of the tree. Phylogenetic analysis of the deduced amino acid sequence of TomLOXF strongly suggests that it belongs to the type-2 family of LOX proteins, within the subgroup including TomLOXC and StLOXH1. Recombinant His-tagged TomLOXF shows 13-LOX activity and uses preferentially linolenate as substrate. Collectively, our data suggest that TomLOXF encodes a 13-LOX probably involved in the production of C6 volatile compounds. This hypothesis is consolidated by the fact that the hydroperoxide lyase of tomato consumes preferentially 13-HPOT [42].

Our transcriptional study of genes coding for five isoforms (*TomLoxA*, *B*, *C*, *D*, and *F*) has revealed that only *TomLoxD *and *TomLoxF *contribute to the enhanced LOX activity observed in *P. putida *BTP1-treated tomatoes.

The time course of *TomLoxD *and *TomLoxF *induction in treated plants compared to controls is very interesting. Levels of transcripts of these genes are higher in bacterized plants during the first days of infection, which are crucial for *B. cinerea *infection of tomato leaves. This early activation of LOX might allow the plant to develop a resistance mechanism during the first stages of disease development. On the other hand, *TomLoxA*, *TomLoxB*, and *TomLoxC *are induced neither by pathogen attack nor by treatment with *P. putida *BTP1. *TomLoxA *is expressed principally in fruits during maturation and in seeds during germination [13]. *TomLoxB *is expressed only in fruits during the latest phase of ripening and during senescence [13]. The absence of stimulation of *TomLoxC *transcription is surprising, as the isozyme encoded by this gene produces hydroperoxides that are consumed principally by the hydroperoxide lyase branch of the oxylipin pathway, and converted into volatiles [14]. Some of these volatiles are fungitoxic [43] or can induce expression of certain genes of the oxylipin pathway, namely *Lox *and *Allene oxide synthase *[44]. *TomLoxC *is stimulated during the early stage of fruit ripening [14], but not by wounding [16].

The stimulation of the linolenate-consuming activity during ISR and *TomLoxF *transcription level are concomitant, suggesting that this isoform contributes to the increased linolenic acid-consuming LOX activity. This gene codes for a protein that consumes linolenic acid preferentially. We showed that it consumes also linoleic acid. But it seems that the increase in linoleic acid-consuming activity caused by the increase of *TomLoxF *transcription is too low to be detectable.

To confirm our results, we also analyzed the accumulation of free 13-HPOT and 13-HOT in plants. 13-HOT, which is produced from 13-HPOT by the hydroperoxyde reductase, by the peroxygenase, or by auto oxidation [45], is more abundant after infection in bacterized plants. 13-HPOT seemed also to be more abundant in *P. putida *BTP1-treated plant than in controls after infection (but the difference was not significant in the second experiment). These results suggest that, after infection, bacterized plants over-produce 13-HPOT by the linolenate-consuming LOX activity, leading to the formation of antifungal 13-HOT. The increase in oxylipin content could also be due to auto oxidation of fatty acid following the pathogen attack, but it can not explain the difference between control and treated plants. Indeed, if the increase was totally caused by auto oxidation after infection, the level of oxylipins should be higher in control plants as the latter show higher infection rates. Only a precedent study showed an oxylipin accumulation in ISR: in bean, *P. putida *BTP1 stimulates the accumulation of 13-HPOT before infection with *B. cinerea*, but the difference was not significative anymore after pathogen challenge [23].

In bean, LOX activity is enhanced before infection and it remains higher in plants treated with *P. putida *BTP1 than in control plants for up to three days after *B. cinerea *inoculation [23]. In cucumber, LOX activity is not stimulated by the rhizobacterium, but the activity of enzymes situated downstream the LOX in the pathway is higher in treated plants during the first days of infection [46]. Hence, stimulation of the oxylipin pathway in the host plant may be a general phenomenon associated with root colonization by *P. putida *BTP1. But if it may be a general phenomenon, it is probably not the only defense mechanism induced in plant by the PGPR. Other defense mechanisms need to be analyzed to determine their implication in *P. putida *BTP1-mediated ISR.

It is interesting to compare our results realized onto whole plants with works realized by Adam et al [24] on the same plant species with the same PGPR and same pathogen, but on cut leaves. With cut leaves, the increase of LOX activity is more rapid in treated plants and, in control and treated tomatoes, reaches its maximal value two days after the beginning of the infection, resulting in higher differences than in our work. In our study, we wanted to see only the effect of *P. putida *BTP1 on whole plant, because the wounding caused by cutting the leaves could interfere with the ISR effect, especially on the LOX, which is induced by wounding [16]. So, it seems important to study defense mechanisms induced by ISR working with non stressed plant material.

## Conclusions

In conclusion, ISR induced by the PGPR *P. putida *BTP1 in tomato is associated with a higher level of *TomLoxD *and *TomLoxF *transcription, the enzyme encoded by the latter gene being a newly identified LOX isoform in this plant. The products of these genes are most probably partly responsible for the increase in overall LOX activity in resistant leaves. LOX might possibly not be the only enzyme of the oxylipin pathway to be stimulated by ISR in tomato. In bean, hydroperoxide lyase is stimulated in response to infection in treated plants [23]. A previous study on detached tomato leaves has revealed that enzymes situated downstream in the LOX pathway are stimulated by *P. putida *BTP1 [24]. Metabolite production and the activities of different enzymes of the oxylipin pathway should be further studied in order to increase our knowledge of the importance of the LOX pathway in ISR.

## Methods

### Microbial strains

*P. putida *BTP1 was selected for its capacity to induce ISR in various plant species (cucumber [22], bean [23], and tomato [24]). This strain was isolated from barley rhizosphere for its ability to produce pyoverdines. *P. putida *BTP1 was maintained on CAA agar medium (5 g/l casamino acids; 0.9 g/l K_2_HPO_4_; 0.25 g/l MgSO_4_.7H_2_O; 15 g/l agar) at 4°C before use. *B. cinerea *was grown on oat-based medium (25 g/l oat flour; 12 g/l agar) at room temperature. The fungus was exposed to UV (15W, at a distance of about 20 cm from the lamp) for one week to induce sporulation.

### Induction of ISR

ISR was induced in tomato (*Lycopersicon esculentum*) cv "merveille des marches", according to the procedure described in [22]. Before sowing, the seeds were rinsed with 0.01 M MgSO_4_.7H_2_O, and soaked for 10 minutes in a bacterial suspension at 10^8 ^CFU ml^-1 ^concentration or, for the control plants, in 0.01 M MgSO_4_.7H_2_O. The seeds were then sown in pots of 10 cm in diameter containing universal compost. The soil was mixed beforehand with a bacterial suspension at 5x10^7 ^CFU g^-1 ^concentration or with an equal volume of 0.01 M MgSO_4_.7H_2_O for untreated plants. The plants were germinated and grown at 26°C, with a 16-h photoperiod (artificial light, with an intensity of 54 μmol.m^-2^.s^-1^). Two and four weeks after sowing, 10 ml of bacterial suspension (concentration: 10^8 ^CFU ml^-1^) were added to the pots of treated plants (and 10 ml of 0.01 M MgSO_4_.7H_2_O to the pots of control plants). After approximately 5 weeks, the tomato plants were transferred to a high-humidity chamber at 20°C, with an 8-h photoperiod. After 24 h, third leaves were infected with *Botrytis cinerea*. Ten 5-μl droplets containing 2500 spores each prepared as described in Ongena et al. [23] were deposited on the adaxial face of each leaf. To determine the infection level, we used a very-used and reproducible phenotypic method [22-24]: 3 days after inoculation of the pathogen, the disease level was determined as the percentage of *B. cinerea *lesions having extended beyond the inoculum drop zone to produce spreading lesions. Three independent experiments were carried out, with 48 plants per treatment. In all these experiments, *P. putida *BTP1-treated plants showed a disease reduction comprised between 33 and 52% compared to controls. The homogeneity of variance for disease reduction evaluation was tested by ANOVA 1 (α = 0.05), and results from the different repetitions were subjected to ANOVA 2 (α = 0.05) so as to pool the data from different experiments.

In every experiment, infected leaves from 12 plants were randomly harvested just before challenge and also 2 days and 4 days after inoculation of the pathogen and immediately frozen in liquid nitrogen. They were then powdered with mortar and pestle and stored at -70°C until used for analyses.

### Methyljasmonate treatment

Five-week-old control tomato plants were transferred to a chamber of 252000 cm^3 ^(20°C, 8-h photoperiod) in the presence of 3 μl methyljasmonate deposited on pads. The following day, leaves were harvested and directly frozen in liquid nitrogen.

### Assay of PAL activity

PAL activity was assayed by monitoring the conversion of L-phenylalanine to L-trans-cinnamate. Powdered frozen leaf tissue (0.25 g) was extracted with 750 ml extraction buffer (50 mM Tris-HCl pH 8.5, 14 mM mercapto-ethanol, 50 g/l polyethylene glycol). Extracts were incubated for 1 h on ice and mixed every 10 minutes by vortexing. Samples were then centrifuged for 10 minutes at 13,000 g at 4°C. The supernatants were collected and PAL activity was determined at 40°C in 3 ml Tris-HCl 50 mM pH 8.5 containing 10 mM L-phenylalanine. Absorbance at 290 nm was monitored for 2 h with a UVIKON XS spectrophotometer (Beun-De Ronde Serlabo sa, Drogenbos, Belgium). The enzymatic activity was calculated using an ε of 11,600 M^-1 ^cm^-1 ^and expressed in nano-enzymatic units per gram fresh weight (μU/g FW).

### Assay of LOX activity

LOX activity was assayed spectrophotometrically. Frozen leaf tissue powder (0.25 g) was added to 750 μl ice-cold 100 mM sodium phosphate buffer containing 0.4 g/l Na_2_S_2_O_5 _and 2.5 g/l Tween 20. The mixture was incubated for 1 hour on ice and mixed every 10 min. After centrifugation (20,000 × g at 4°C for 10 min), 20 μl of the supernatant was added to 976 μl of oxygenated 100 mM sodium phosphate buffer pH 7.0 and 4 μl of 18 mM linolenic or linoleic acid in 0.05N NaOH. Enzymatic activity was determined by monitoring the appearance of hydroperoxides at 234 nm at 30°C for 15 min with a UVIKON XS spectrophotometer. LOX activity was calculated using an ε of 25,000 M^-1 ^cm^-1 ^and was expressed in micro-enzymatic units per gram fresh weight (mU/g FW). The activity assay was performed with leaf material collected at each time point. For the proteins extracted from *E. coli*, the LOX activity was tested by using 20 μl of total or semi-purified proteins, 976 μl of oxygenated 100 mM sodium phosphate buffer pH 7 and 4 μl of 18 mM linolenic or linoleic acid in 0.05N NaOH.

### Cloning of the *TomLoxC *and *TomLoxF *cDNA probes

For the synthesis of the *TomLoxC *cDNA, total RNA from leaves of tomato treated with *Bacillus subtilis *M4 was used. *TomLoxF *cDNA was first obtained from total RNA of methyljasmonate-treated tomato leaves. First-strand cDNA was synthesized (Smart PCR cDNA Synthesis Kit, Clontech, Saint-Germain-en-Laye, France) according to the manufacturer's instructions. PCR amplifications were performed with the Advantage 2 PCR kit (Clontech). PCR reactions contained 1 μl first-strand cDNA and each primer at 0.5 μM. The primers used, designed on the basis of the *TomLoxC *cDNA sequence (GenBank: U37839), were TomLoxC-1F (5'-ATCCTAGAAGGTGTAGAACCGGTC-3') and TomLoxC-1R (5'-TGATTCTGGAGGTCCAGACAC-3'). Samples were amplified in a GeneAmp 9700 PCR System (Perkin-Elmer) as follows: 3 min at 94°C; 30 cycles of 1 min at 94°C, 1 min at 58°C, and 1 min at 72°C; 7 min at 72°C. The PCR products were cloned by TA-cloning in pGEM-T Easy vectors (Promega Corp., Madison, WI, U.S.A.), according to the manufacturer's protocols. Plasmids showing inserts of the expected size after restriction were selected and sequenced by CoGenics (Grenoble, France).

### RACE-PCR of *TomLoxF*

To obtain the 5'- and 3'-ends of the coding sequence of *TomLoxF*, rapid amplification of cDNA ends (RACE) was performed with the SMART RACE cDNA Amplification Kit (Clontech) according to the manufacturer's instructions. The 3'-RACE Ready and 5'-RACE Ready first-strand cDNAs were synthesized from 1 μg total RNA extracted from leaves of tomato plants treated with methyljasmonate or *P. putida *BTP1. Subsequent rapid amplification of cDNA ends by PCR was then performed with the 3'-RACE (*TomLoxF *3Ra 5'-AGGGTTGCAATCGATCATCACAGACCG-3') and 5'-RACE (*TomLoxF *5Ra 5'-TGAAGTGCCGGAAGTTCTACTTCGACG-3') gene-specific primers. *TomLox*F gene-specific primers were designed on the basis of the partial sequence of *TomLoxF *obtained as described above. They were designed to allow the use of touchdown PCR, which significantly improves the specificity of SMART RACE amplifications. Touchdown PCR was performed as recommended by the manufacturer. The 5'- and 3'-RACE products were cloned into the pGEM-T Easy vector (Promega), and sequenced by CoGenics.

### RNA gel blot hybridizations

Total mRNA was extracted from frozen leaf tissue powder by the phenol/SDS method described by Ausubel et al. [47]. Analysis of gene expression was done by hybridization on Northern blots. RNA (20 μg) was electrophoresed through a formaldehyde agarose gel and blotted onto Hybond N+ membranes (Amersham, Little Chalfont, UK). Equal lane loading was checked by visualizing ethidium-bromide-strained ribosomal RNA after electrophoresis. The membrane was hybridized with DNA probes labeled by random priming with [α-^32^P]dATP according to the procedure recommended by the manufacturer (Random Primers DNA labeling System; Invitrogen, Carlsbad, Calif.). Blots were hybridized with the following probes: a 904-bp *Xba*I fragment of the *TomLoxA *cDNA [13], a 855-bp *HindIII *fragment of the *TomLoxB *cDNA [13], a 506-bp *Eco*RI fragment of the *TomLoxC *cDNA [16], a 910-bp *Bgl*II fragment of the *TomLoxD *cDNA [16], and a 500-bp *Eco*RI fragment of the *TomLoxF *cDNA. After hybridization, the blots were washed and used to expose X-Ray film (Fujifilm, Japan) for at least 48 h.

### Bioinformatic analysis

Homology analysis of the cDNA and deduced amino acid sequences of *TomLoxF *were performed with the Blastp 2.2.19+ and Blastn 2.2.19+ programs, available from the National Center for Biotechnology Information http://blast.ncbi.nlm.nih.gov/Blast.cgi. ClustalW2 [48,49] was used to align the deduced TOMLOXF protein sequence with the sequences of other plant LOX proteins and an unrooted tree was constructed, with the help of the TreeDyn program [50,51], publicly available at the site phylogeny.fr [52,53]. Subcellular localization was predicted with four different programs: ChloroP 1.1 [54,55], WoLF PSORT [56] and iPSORT Prediction [57,58]. Conserved domains in the deduced amino acid sequences were determined by the NCBI Conserved Domain Search program [59]. For all the bioinformatic analyses, default settings were used.

### *TomLoxD *and *TomLoxF *genes amplification and cDNA isolation for expression

The cDNA sequence of *TomLoxD *and *TomLoxF *was synthesized by RT-PCR from total RNA of *Lycopersicon esculentum *c. "merveille des marchés" by using couples of primer 1(CATGCCATGGGTCACCACCACCACCACGCTATAAGTGAAAATTTGGTCAAAGTTGTG) and primer 2 (CCGCTCGAGTTATATCGATACACTATTTGGAAC) - primer 3 (CATGCCATGGCAGCTATAAGTGAAAATTTGGTCAAAGTTGTG) and primer 4 (CCGCTCGAGTTATATCGATACACTATTTGGAAC) - primer 5 (CATGCCATGGGTGCTGTAGTTACAGTAAGGAAC) and primer 6 (CCGCTCGAGTATCGATACACTATTTGGAAC) - primer 7 (CATGCCATGGGTCACCACCACCACCACATGGCACTTGCTAAAGAAATTATG) and primer 8 (CCGCTCGAGTTATATCGATACACTATTTGGAAC) for *TomLoxD*; and primer 1 (5'-CTAGCTAGCAGTTCTACTGAAAATTCCTC-3') and primer 2 (5'- CCGCTCGAGTTAAATGGAAATGCTATAAGGTAC-3') for *TomLoxF*. These primers were designed to exclude of the predicted chloroplastic peptide signal from the amplification product. Artificial restriction sites were introduced for NcoI and XhoI respectively in primers 1, 3, 5, 7 and primers 2, 4, 6, 8 for TomLoxD (underlined) and for *Nhe*I and *Xho*I respectively in primer 1 and 2 for *TomLoxF *(underlined) in order to clone the product in the pET28-a plasmid. For the reverse transcription step 1 μg of total RNA was denatured 5 min at 70°C in presence of 0.5 μM primer 1 and 1× RNase OUT (Biolabs) in a total volume of 12 μl. After cooling for 5 min at 4°C, the mix was incubated in 1× M-MLV buffer with 10 mM DTT, 0.5 mM dNTPs and 200 U M-MLV, for 1 h at 42°C and then 15 min at 70°C. 5 μl of the reverse transcription products, in presence of 0.5 mM dNTPs, 0.5 μM of each primer and 2 U of DNA Taq polymerase, were used for the PCR. Samples were amplified in a GeneAmp 9700 PCR System (Perkin-Elmer) as follows: 5 min at 94°C; 10 cycles of 1 min at 94°C, 1 min at 56°C, and 4 min at 72°C; 30 cycles of 1 min at 94°C, 1 min at 66°C, and 4 min at 72°C; 7 min at 72°C.

### Expression of *TomLoxD *and *TomLoxF *in *E. coli*

The *TomLoxD *and *TomLoxF *cDNA fragment were digested by respectively by *Nco*I-*Xho*I and *Nhe*I-*Xho*I, and purified using the Nucleospin extract II kit (Macherey-Nagel). The fragment was ligated into the expression plasmid pET28-a previously digested by NcoI-XhoI for *TomLoxD *and *Nhe*I-*Xho*I for *TomLoxF *and purified. As a result, the *TomLoxD *and *TomLoxF *cDNA were cloned dowstream of the T7 promoter of the T7 phage. *E. coli *BL21 strain was transformed with pET28-a containing or not the *TomLox *sequence by electroporation (2.5 kV/cm, 25 μF and 100 Ω). Transformants were selected on LB medium containing 50 μg/ml kanamycin. Plasmid DNA was extracted with Nucleobond Xtra Midi columns (Macherey-Nagel) and the 5' part of *TomLoxF *was analyzed by sequencing to confirm the correct integration of the fragment.

A freshly grown colony was inoculated in 2 ml of LB medium containing 50 μg/ml kanamycin and grown overnight at 37°C. 50 μl of this preculture was transferred to 50 ml of LB medium containing kanamycin and grown at 37°C. When the O.D. reached 0.8, temperature was dropped down to 18°C and 1 mM isopropyl β-D-1-thiogalactopyranoside (IPTG), was added in the medium to induce the transcription of *TomLoxD *and *TomLoxF*. After 20 h of induction, the culture was centrifuged at 3500 rpm at 4°C and the supernatant was eliminated. The cell pellet was suspended in 50 mM sodium phosphate buffer (1/10 of culture volume) containing 1 mM PMSF and sonicated (75 W) 6 times during 30 s (Vibra Cell, Sonics and Materials), and then centrifuged 2 times 10 min at 13,000 g at 4°C. The supernatant containing the proteins was conserved.

### SDS-PAGE analysis and immunodetection

SDS-PAGE was performed as described by Laemmli [60] in gels containing 10% acrylamide. Protein bands were visualized by staining with Coomassie blue: the gel was soaked for 1 h in Coomassie blue (Code Blue Stain Reagent (Thermo Scientific)) and washed over night in water. Proteins contained in the gel were transferred onto a nitrocellulose membrane (GE Healthcare) using a Multiphor II apparatus (GE Healthcare). After blocking for 16 h in TBS buffer (50 mM Tris, 150 mM NaCl, pH 7,5) containing 1× blocking reagent (Roche), the membrane was incubated for 1 h with the primary anti-pentahistidin monoclonal antibody (Chemicon International) in TBS. Then the membrane was washed four times for 10 min in TBS, incubated 30 min with GtxMs IgG (Chemicon International) in TBS, and washed four times for 10 min in TBS. Finally proteins exhibiting a pentahistidine tail were detected with the BM Chemiluminescence Western Blotting Kit (Roche) according to the manufacturer's instructions. After this last stage, the blot was exposed to a X-Ray film (Fujifilm, Japan) for 5 min.

### Purification of His-tagged proteins

His-tagged proteins were purified by nickel column affinity chromatography (Äkta, Amersham Pharmacia Biotech) according the method described in Boutaud and Brash [61]. The supernatant containing the proteins was loaded on a nickel column (Hightrap HP, 1 ml, Amersham Pharmacia Biotech) equilibrated with 50 mM potassium phosphate buffer, pH 7.2, 500 mM NaCl at 1 ml/min. The column was then washed with the equilibration buffer and the nonspecific bound proteins were eluted with 50 mM potassium phosphate buffer, pH 7.2, 500 mM NaCl, 500 mM glycine. His-tagged proteins were eluted with 50 mM potassium buffer, pH 7.2, 500 mM NaCL, 40 mM L-Histidine. Fractions of 1,5 ml were collected and analyzed by SDS-PAGE, Western blotting and immunodetection. LOX activity was also assayed in the different fractions after 15 fold concentration on a vivaspin II column (Sartorius Stedim Biotech).

### Determination of free 13-HPOT and 13-HOT concentrations

Oxylipins were extracted from plant tissues and quantified by HPLC using a method described in Fauconnier et al.[62]. 1 g of powdered frozen leaf tissue was extracted in 20 ml of extraction medium (isohexane/2-propanol, 3/2 (v/v), 0.0025% (w/v) butylated hydroxytoluene). (6Z, 9Z, 11E, 13S)-13-hydroxy-6, 9, 11-octadecatrienoic acid was used as internal standard. After homogenization, the extract was centrifuged at 1,300 g at 4°C for 10 min. The upper phase was collected and added to a 6,7% (w/v) solution of potassium sulfate to reach a volume of 32.5 ml. After 10 min of shaking at 4°C, the extract was centrifuged at 1,300 g at 4°C for 10 min. The upper phase was collected. HPLC analysis was performed in two steps. The first step was carried out on a reverse phase column, allowing the recuperation of free oxylipins. The fraction of interest was collected and then injected on straight-phase column, to separate the 13-HPOT and 13-HOT. For the reverse phase step, a volume of 80 μl of sample was injected onto an EC250/2 Nucleosil 120-5 C18 column (250 × 2.1 mm, 5 μm particle size, Macherey and Nagel, Easton, PA, USA) using this gradient system: solvent A (methanol/water/acetic acid (75:15:0,1) (v/v)) and solvent B (methanol/water/acetic acid (100:0:0,1) (v/v)) according to this gradient program: 20% solvent B for 10 min, followed by a linear increase of solvent B up to 40% within 28 min, then a linear increase of solvent B up to 100% within 30 min and held for 15 min, then a linear decrease up to 20% solvent B within 5 min and finally an isocratic post-run at 15% solvent B for 6 min. The flow rate was 0.18 ml/min up to 30 min and increased linearly to 0.36 ml/min within 35 min, held for 10 min, followed by a linear decrease to 0.18 ml/min within 50 min and post-run for 6 min. Straight-phase HPLC was performed on a Zorbax Rx-SIL column (150 × 2.1 mm, 5 μm particule size, Agilent, Palo Alto, CA, USA) with n-hexane/2-propanol/acetic acid (100:1:0.1 (v/v/v)) as a solvent system at a flow rate of 0.2 ml/min. 25 μl of sample were injected and the absorbance was recorded at 234 nm. The results were expressed in nmol/g FW. The analysis was performed in duplicate.

To determine the products of the reaction catalyzed by the recombinant TOMLOXF, we synthesized hydroperoxides from fatty acids according to a method described in Royo et al [25]. Total proteins were extracted from *E. coli *expressing TOMLOXF as described above. Linoleic and linolenic acids (25 μmol), in combination or separated, were incubated with the protein extract (containing 6,032 U of LOX activity) in 25 ml of oxygen-saturated 50 mM sodium phosphate buffer pH 6. After incubation at 25°C for 15 min with a constant flow of oxygen, the produced hydroperoxides were precipitated by lowering the pH to 3.0 with 6 N HCl. Then, we purified and analyzed the produced hydroperoxides on a C18 microcolumn (500 mg) according a method used by Fauconnier et al [63]. The LOX reaction products were separated and analyzed by HPLC with a Inertsil 5 ODS 2 250 × 4,6 mm (Chromasil) column with water, acetonitril and NaH_2_PO_4 _as solvant (45:45:10 v/v during 22 min, then 37:53:10 v/v for 42 min, and 37:53:10 v/v until 120 min), flow rate of 0.5 ml/min, detection at 234 nm. The retention time of TOMLOXF hydroperoxides were compared with those of commercial 13- and 9- HPOD/HPOT (Larodan).

## List of abbreviations

ISR: induced systemic resistance; HOD: hydroxy octadecadienoic acid; HOT hydroxy octadecatrienoic acid; HPOT: hydroperoxy octadecatrienoic acid; LOX: lipoxygenase; HPOD: hydroperoxy octadecadienoic acid; PAL: phenylalanine ammonia-lyase; PGPR: Plant Growth Promoting Rhizobacteria; SAR: systemic acquired resistance.

## Authors' contributions

MM carried out the culture and treatment of tomatoes, enzymatic and gene expression analysis, cloning and expression of *TomLoxD *and *TomLoxF *in *E. coli*, purification of the protein and determination of its substrates and products, statistic analysis and wrote the manuscript. FD and AA realized *TomLoxC *and *TomLoxF *probes. FD and CB realized RACE-PCR. FD realized bioinformatic analysis of TOMLOXF. FD, MO, MLF, PT and JD conceived the study, participated in its design and coordination and helped to draft the manuscript. All authors read and approved the final manuscript.
